# Effects of white-coat, masked and sustained hypertension on coronary artery stenosis and cardiac arrhythmia

**DOI:** 10.1038/s41440-019-0342-3

**Published:** 2019-10-17

**Authors:** Peng Cai, Weitian Zhong, Yan Wang, Xukai Wang

**Affiliations:** 1 Department of Cardiology, Institute of Field Surgery, Daping Hospital, Army Medical University, Chongqing, China; 2 Key Laboratory of Basic Pharmacology of Ministry of Education and Joint International Research Laboratory of Ethnomedicine of Ministry of Education, Zunyi Medical University, Zunyi, China

**Keywords:** White-coat hypertension, Masked hypertension, Sustained hypertension, Coronary artery stenosis, Cardiac arrhythmia

## Abstract

This study aimed to investigate whether hypertension phenotypes such as white-coat hypertension (WCHT), diagnosed with the addition of nighttime blood pressure (BP) criteria, are related to coronary artery stenosis (CAS) and cardiac arrhythmia. In this cross-sectional observational study, 844 participants who did not use antihypertensive, lipid-lowering, and antiplatelet drugs were selected. The subjects were divided into normotensive (NT), WCHT, masked hypertension (MHT), and sustained hypertension (SHT) groups based on the results of clinic BP measurement and ambulatory BP monitoring. Coronary angiography and ambulatory electrocardiography were performed to determine the participants’ CAS and cardiac arrhythmia status. Coronary angiography revealed 556 patients with CAS and 288 participants with normal coronary arteries. The chi-squared test showed that the incidence of CAS was higher in the MHT and SHT groups than in the NT group, while no significant change was found in the WCHT group (*P* = 0.003, *P* < 0.001, *P* = 0.119). The logarithm of the Gensini score was used to compare the degree of CAS between the groups. Multiple linear regression analysis showed that the degree of CAS was higher in the WCHT, MHT, and SHT groups than in the NT group (*P* < 0.05). The incidences of frequent atrial premature beats, atrial tachycardia, and ventricular cardiac arrhythmia were significantly higher in the WCHT and SHT groups than in the NT group, while only ventricular cardiac arrhythmia changes were observed in the MHT group. This study found that hypertension phenotypes such as WCHT were closely associated with CAS and cardiac arrhythmia.

## Introduction

Hypertension diagnosed only by clinic blood pressure (BP) measurement cannot explain many clinical phenomena. Hypertension phenotypes such as white-coat hypertension (WCHT), masked hypertension (MHT), and sustained hypertension (SHT) classified by clinic BP measurement and ambulatory BP monitoring can facilitate an in-depth understanding of the hypertension-mediated organ damage [1, 2]. Among the hypertension phenotypes, the cardiovascular damage of SHT is well established, and that of MHT is gradually becoming clearer, while the cardiovascular damage of WCHT remains controversial [3]. The meta-analysis results of Huang et al. in 2017 showed that WCHT significantly increased the incidence of cardiovascular events and total mortality [4]. An analysis of a large ambulatory BP database showed that WCHT was associated with an increased risk of hospitalization for hypertensive crisis and heart failure [5]. Takeshi Fujiwara et al. also found that the risks for cardiovascular outcome and deterioration to SHT were significantly higher in individuals with WCHT [6]. WCHT appears to cause cardiovascular damage [7]. However, most previous studies used the traditional diagnostic criteria, that is, only relying on daytime ambulatory BP monitoring or 24-h ambulatory average BP to define hypertension phenotypes such as WCHT, without considering the impact of nocturnal hypertension on the hypertension phenotypes classification [8–10]. The 2014 European Society of Hypertension practice guidelines proposed a classification method combining daytime BP, nighttime BP, 24-h BP, and clinic BP to classify hypertension phenotypes, which is accepted worldwide [11]. Under the new diagnostic criteria, the distribution of hypertension phenotypes has changed significantly. Some patients with WCHT were reclassified as having SHT, while some with normal BP were redefined as having MHT. De La Sierra et al. found that WCHT accounted for 41.3% of clinic hypertension according to the traditional criteria for classifying hypertension phenotypes, as compared with 26.1% according to new criteria [12]. The results of the Spanish Registry and Jackson Heart Study also showed that the prevalence of WCHT was dependent on definition criteria [13]. Asayama et al., using the IDACO database, found both WCHT defined by normal 24-h BP and WCHT defined by normal daytime BP had cardiovascular effects. Only diagnostic criteria that considered the normality of all ambulatory periods identified patients with cardiovascular risk similar to that of normotensive (NT) patients; however, cardiovascular risk factors such as elevated cholesterol and body mass index (BMI) still existed in WCHT defined by normality of all periods [14]. The cardiovascular risk of WCHT was significantly reduced after adding nighttime diagnostic criteria. Whether WCHT defined by normality of all periods has cardiovascular damage is unclear. With the significant proportion changes in MHT, the cardiovascular damage of MHT also needs to be further explored.

At present, the clinical studies of hypertension phenotypes mainly focus on the ventricular structure, but the effect on coronary artery stenosis (CAS) is seldom studied, and the influence on cardiac arrhythmia has not yet been explored [15]. Dimitrios et al. found that WCHT can increase CAS under the traditional cutoff criteria, but whether WCHT has an injurious effect on coronary arteries after adding the nighttime diagnostic criteria remains unknown [16]. The relationship between MHT and CAS has not been previously reported. Atrial and ventricular arrhythmias are important manifestations of cardiac target organ injury and are closely related to cardiovascular events such as coronary heart disease and sudden cardiac death [17]. Hypertension is significantly associated with increased atrial and ventricular arrhythmias, and the mechanism may be related to changes in cardiac structure [18]. Previous studies have shown that WCHT, MHT, and SHT increase the left ventricular mass index and alter the cardiac structure [19, 20]. Whether WCHT and MHT trigger cardiac arrhythmias remains unknown. Based on these findings, we designed a clinical study to explore whether WCHT and other hypertension phenotypes can cause CAS and increase the degree of CAS after adding nighttime diagnostic criteria. We also explored the relationship between atrial and ventricular arrhythmias and hypertension phenotypes such as WCHT.

### Participants and methods

#### Study participants

Between December 2017 and April 2019, 2106 participants with suspected coronary artery disease were enrolled at the Daping Hospital of the Army Military Medical University. Inclusion criteria were adults with symptoms of angina and electrocardiographic changes in myocardial ischemia. We also screened the patients according to the following exclusion criteria: (1) antihypertensive drug users; (2) use of lipid-lowering and antiplatelet drugs; (3) secondary hypertension, such as renal parenchymal and renovascular hypertension, primary aldosteronism, pheochromocytoma, and Cushing syndrome; (4) renal insufficiency or hepatic insufficiency (alanine aminotransferase ≥ 80 IU/L, serum creatinine ≥ 133 μmol/L); (5) congenital heart disease, valvular heart disease, and cardiomyopathy; and (6) previous coronary artery bypass grafting or coronary stent implantation. A total of 435 males and 409 females were included in the final samples, with an average age of 61.7 ± 10.6 years. The study was reviewed and approved by the Ethics Committee of Daping Hospital and registered in the Chinese Clinical Trial Registry (registration number: ChiCTR1800015507). The participants provided informed consent for this study.

### Study grouping

The participants were grouped based on the results of clinic BP measurement and ambulatory BP monitoring according to the European Hypertension Practice Guide 2018 [21]. The threshold of elevated clinic BP was ≥140/90 mmHg. The mean ambulatory BP was ≥135/85 mmHg in the daytime, ≥120/70 mmHg at night, and ≥130/80 mmHg during the whole day. The four groups were as follows: SHT, participants with elevated clinic and ambulatory BPs; WCHT, participants with elevated clinic BPs, and normal ambulatory BPs; MHT, participants with normal clinic BPs, and elevated ambulatory BPs; and NT, participants with normal clinic and ambulatory BPs [22]. In addition, we grouped the participants according to previous criteria to show changes in the proportion of hypertension phenotypes. Previous diagnostic criteria A: phenotypic classification was based on the clinic BP and daytime BP. Elevated clinic BP was ≥140/90 mmHg, and the standard for ambulatory BP was ≥135/85 mmHg in the daytime. Diagnostic criteria B: phenotypic classification was based on the clinic BP and daytime BP. Elevated clinic BP was ≥140/90 mmHg, and the mean ambulatory BP was ≥130/80 mmHg during the whole day [12, 23].

### General data

The general data were collected using questionnaires and included age, gender, smoking history, drinking history, and diabetes mellitus. The height and weight of the participants were measured in the field, and the BMI was calculated. BMI = weight (kg) ÷ height^2^ (m).

### BP measurement

After 20 min of sitting and rest, the right brachial artery BP was measured by medical staff using a mercury sphygmomanometer. The mean values of three different BP measurements were taken as the clinic BP. The interval between each BP measurement was ≥12 h. The 24-h ambulatory BP monitoring was conducted using the ambulatory electrocardiogram (ECG) BP recorder CB-2301-A (Wuxi, China). The BP between 6:00 and 22:00 was measured as daytime BP and that between 22:00 and 6:00 as the nighttime BP. The patients were asked to work and rest according to this rule. The BP was measured every 30 min in the daytime, and the effective daytime BP count was required to be >80%. The nighttime BP was required to have one effective BP measurement per hour. The participants were instructed to avoid unusual physical activities and to keep their arm still during BP measurements. Average values for the 24-hour, daytime, and nighttime systolic and diastolic BP levels and heart rate were calculated. The values of 24-h SBP standard deviation, 24-h DBP standard deviation, 24-h SBP coefficient of variation, and 24-h DBP coefficient of variation parameters were obtained by analyzing the variability of the ambulatory BP monitoring results.

### ECG examination

A 24-h ambulatory ECG examination was performed using the ambulatory ECG BP recorder CB-2301-A (Wuxi, China). Heart rate variability was analyzed by time domain analysis using the ambulatory ECG analysis software V6.1.9 (Wuxi, China). The values of standard deviation of normal-to-normal RR intervals (SDNN), mean standard deviation of RR intervals (MSD), root mean square of successive differences between adjacent RR intervals (RMSSD), and percentage of adjacent NN intervals differing by >50 ms (pNN50) parameters reflecting the cardiac autonomic nervous system were obtained [24]. The increase in SDNN and PNN50 reflected the weakening of cardiac sympathetic function, and the increase in RMSSD and MSD reflected the strengthening of cardiac vagus nerve function [25]. The results of 24-h ambulatory ECG recording were interpreted by experienced technicians to identify frequent atrial premature beats, atrial tachycardia, atrial fibrillation, and various ventricular arrhythmias.

### Biochemical tests

Venous blood was collected after 12 h of fasting, and total bilirubin, alanine aminotransferase, aspartate aminotransferase, total cholesterol, triglyceride, high-density lipoprotein cholesterol, low-density lipoprotein cholesterol (LDL-C), apolipoprotein B, fasting blood glucose, serum creatinine, and uric acid levels were detected by a BECKMAN DXC800 automatic biochemical analyzer (Brea, USA). Hyperlipidemia and hyperuricemia were diagnosed according to the test results. Hyperlipidemia was defined as total cholesterol ≥ 5.18 mmol/L, triglyceride ≥ 1.70 mmol/L, or LDL-C ≥ 3.37 mmol/L. Hyperuricemia was defined as blood uric acid > 420 μmol/L [26, 27].

### Coronary arteriography

Trained cardiologists performed coronary angiography to distinguish patients with CAS from those with normal coronary arteries. The Gensini scoring system was used to assess the severity of CAS after arteriography [28]. The lumen diameter decreased by 1–25%, 26–50%, 51–75%, 76–90%, 91–99%, and 100%, respectively, with scores of 1, 2, 4, 8, 16, and 32. The left main coronary artery lesion coefficient was 5 when the fraction of stenosis was multiplied by the coefficients of different lesion sites. The proximal segments of the left anterior descending branch and the circumflex branch had a lesion coefficient of 2.5. The lesion coefficient of the middle segment of the left anterior descending artery was 1.5. The distal segment of the left anterior descending branch, the first diagonal branch, the middle and distal segment of the circumflex branch, the obtuse marginal branch, the right coronary artery and the right posterior descending branch were rated as 1 point. The lesion coefficient of the second diagonal branch, right posterior coronary artery and other small branches was 0.5. The severity of CAS was expressed as the sum of all vascular lesion scores for each patient [29].

### Statistical analysis

The data were recorded by Epidata 3.1 software (Odense, Denmark) and analyzed by SPSS 22.0 software (New York, USA). Pearson’s chi-squared test was used to explore the difference in the proportions among the groups. One-way ANOVA was used to analyze the differences between groups if the measured data satisfied the homogeneity of variance and normal distribution, and the Kruskal–Wallis rank sum test was used if the measured data did not satisfy the homogeneity of variance or normal distribution. The Gensini score was logarithmic transformed to Lg (Gensini score), which satisfies the homogeneity of variance and normal distribution. Unconditional logistic regression analysis was used to investigate the risk factors of CAS and determine the association between arrhythmias and hypertension phenotypes after adjusting for confounding factors such as age, gender, BMI, smoking history, drinking history, diabetes mellitus, hyperuricemia, and hyperlipidemia. Forward options were used to select the significant covariates, and the goodness of fit of logistic regression models was tested by the Hosmer–Lemeshow test. Multiple linear regression analysis was used to determine the association between Lg (Gensini score) and hypertension phenotypes after adjusting for age, gender, BMI, smoking history, alcohol consumption history, and the presence of diabetes, hyperuricemia, or hyperlipidemia. The graphs were generated by GraphPad Prism 8.0 software (La Jolla, USA).

## Results

**Fig. 1:**
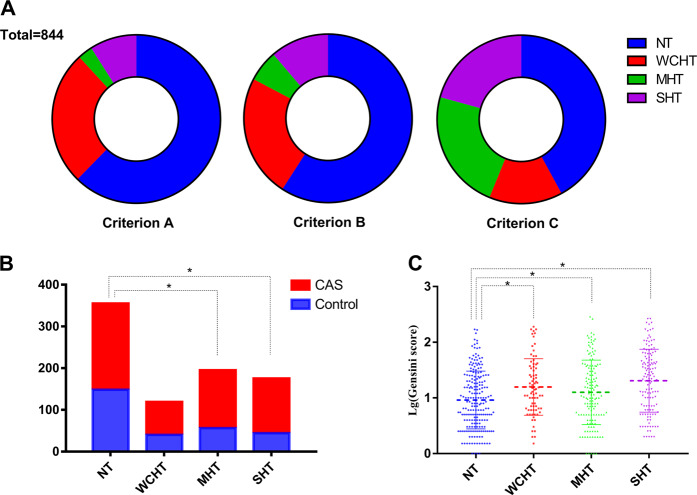
**a** Proportion of hypertension phenotypes under different diagnostic criteria. **b** The incidence of coronary artery stenosis in different hypertension phenotypes. **c** The degree of coronary artery stenosis in different hypertension phenotypes

This was a cross-sectional observational study. Between January 2018 and April 2019, 2106 adults volunteered to participate in the study. After screening the participants’ medical history and examination results, 923 patients were excluded for antihypertensive drug use, 232 patients for antiplatelet or statin drug use, nine patients for secondary hypertension, 48 patients for hepatorenal insufficiency, 18 patients for congenital heart disease, valvular heart disease or cardiomyopathy, and 32 patients for previous coronary stenting or coronary bypass grafting. A total of 844 participants were finally included in the study. There were 355 (42.1%) patients in the NT group, 119 (14.1%) patients in the WCHT group, 195 (23.1%) patients in the MHT group, and 175 (20.7%) patients in the SHT group. Of these, 426 cases (62.3%) in the NT group, 218 cases (25.8%) in the WCHT group, 24 cases (2.8%) in the MHT group, and 76 cases (9.0%) in the SHT group were diagnosed by conventional diagnostic criteria A, while 499 cases (59.1%) in the NT group, 199 cases (23.6%) in the WCHT group, 51 cases (6.0%) in the MHT group, and 95 cases (11.3%) in the SHT group were diagnosed by diagnostic criteria B. Significant changes in the percentage of hypertension phenotypes are noted in Fig. 1a.

### General information and biochemical results

After addition of the diagnostic criteria for nighttime BP, there was no significant difference in gender among the four groups, but the age of the WCHT group was higher than that of the NT group (64.3 ± 10.1 vs 60.4 ± 10.6, *P* *<* 0.001, Table 1). The BMI in the MHT and SHT groups was significantly higher than that in the NT group (24.0 ± 3.1 vs 23.2 ± 3.1, *P* = 0.002; 25.1 ± 3.6 vs 23.2 ± 3.1, *P* < 0.001; Table 1). The plasma glucose concentration in the WCHT, MHT, and SHT groups was higher than that in the NT group (*P* < 0.001, *P* = 0.006*, P* < 0.001; Table 1). There was a significant difference in triglyceride and apolipoprotein B levels between the WCHT group and the NT group (1.9 ± 1.3 vs 1.6 ± 1.0 mmol/L, *P* = 0.007; 0.9 ± 0.3 vs 0.9 ± 0.2 g/L, *P* = 0.005; Table 1), while the total cholesterol, triglyceride, and LDL-C were higher in the MHT and SHT groups than in the NT group (*P* < 0.05; Table 1). Renal function tests showed that serum uric acid in the SHT group was higher than that in the NT group (343.9 ± 90.7 vs 313.7 ± 83.7 μmol/L, *P* < 0.001, Table 1). The detailed results are shown in Table 1.

**Table 1:** General characteristics of participants stratified into four BP categories

Parameters	NT	WCH	MHT	SHT	*P* value
Individuals (%)	355 (42.1)	119 (14.1)	195 (23.1)	175 (20.7)	–
Female (%)	181 (51.0)	61 (51.3)	95 (48.7)	106 (41.1)	0.169
Age (y)	60.4 ± 10.6	64.3 ± 10.1*	62.2 ± 10.5	62.2 ± 10.8	0.003
BMI (kg/m2)	23.2 ± 3.1	23.8 ± 3.0	24.0 ± 3.1*	25.1 ± 3.6*	<0.001
Family history (%)	52 (14.6)	28 (23.5)	30 (15.4)	45 (25.7)	0.005
Smoking (%)	99 (27.9)	37 (31.1)	66 (33.8)	61 (34.9)	0.315
Drinking (%)	84 (23.7)	30 (25.2)	42 (21.5)	45 (25.7)	0.792
Diabetes (%)	40 (11.3)	17 (14.2)	35 (17.5)	29 (16.6)	0.138
Hyperlipidaemia (%)	175 (49.3)	65 (54.6)	104 (53.3)	113 (64.6)	0.011
Hyperuricemia (%)	37 (10.4)	15 (12.6)	26 (13.3)	34 (19.4)	0.040
Total bilirubin (μmol/L)	12.5 ± 5.1	12.7 ± 5.3	12.3 ± 5.0	12.8 ± 4.7	0.750
Alanine aminotransferase (IU/L)	22.2 ± 13.1	23.9 ± 15.0	23.1 ± 13.3	26.9 ± 17.5	0.075
Aspartate aminotransferase (IU/L)	23.9 ± 11.4	25.7 ± 13.5	24.6 ± 11.8	27.8 ± 15.8	0.072
Total cholesterol (mmol/L)	4.3 ± 1.0	4.5 ± 1.1	4.5 ± 1.1*	4.5 ± 1.0*	0.042
Triglyceride (mmol/L)	1.6 ± 1.0	1.9 ± 1.3*	1.8 ± 1.3*	2.1 ± 1.6*	<0.001
High-density lipoprotein cholesterol (mmol/L)	1.2 ± 0.3	1.2 ± 0.3	1.1 ± 0.3	1.1 ± 0.2	0.145
Low-density lipoprotein cholesterol (mmol/L)	2.7 ± 0.7	2.8 ± 0.7	2.9 ± 0.8*	2.9 ± 0.8*	0.042
Apolipoprotein B (g/L)	0.9 ± 0.2	0.9 ± 0.3*	0.9 ± 0.3	0.9 ± 0.3*	0.012
Glucose (mmol/L)	5.1 ± 1.4	5.8 ± 2.4*	5.8 ± 4.5*	5.6 ± 1.6*	<0.001
Serum creatinine (μmol/L)	65.7 ± 14.3	66.4 ± 16.1	78.8 ± 58.9	68.8 ± 16.0	0.322
Uric acid (μmol/L)	313.7 ± 83.7	323.9 ± 93.5	326.3 ± 85.4	343.9 ± 90.7*	0.003
Coronary artery stenosis (%)	207 (58.3)	79 (66.4)	139 (71.3)	131 (74.9)	<0.001

*NT* normotension, *WCHT* white-coat hypertension, *MHT* masked hypertension, *SHT* sustained hypertension, *CAS* coronary artery stenosis

*P < 0.05

### Blood pressure (BP)

BP parameters such as clinic BP and the average values for the 24-h, daytime, and nighttime BP levels are presented in Table 2. The clinic systolic BP in the WCHT, MHT, and SHT groups was higher than that in the NT group (143.2 ± 12.6 vs 113.3 ± 11.7 mmHg, *P* < 0.001; 117.7 ± 11.8 vs 113.3 ± 11.7 mmHg, *P* = 0.003; 149.2 ± 16.3 vs 113.3 ± 11.7 mmHg, *P* < 0.001, Table 2). The clinic diastolic BP in the WCHT and SHT groups was higher than that in the NT group, but there was no change in the MHT group (83.9 ± 10.7 vs 71.5 ± 8.2 mmHg, *P* < 0.001; 90.2 ± 13.3 vs 71.5 ± 8.2 mmHg, *P* < 0.001; 72.7 ± 8.4 vs 71.5 ± 8.2 mmHg, *P* = 0.154, Table 2). The 24-h BP and daytime BP were significantly different among the three groups (*P* < 0.01, Table 2). The nocturnal systolic BP and diastolic BP were higher in the MHT and SHT groups compared with the NT group, while only the nocturnal systolic BP was higher in the WCHT group (*P* < 0.01, Table 2).

**Table 2:** Blood pressure and heart rate levels of participants stratified into four BP categories

Parameters	NT	WCHT	MHT	SHT	*P* value
Clinic BP measurement					
Systolic BP (mmHg)	113.3 ± 11.7^#,△,▽^	143.2 ± 12.6*^,△^	117.7 ± 11.8*^,△,▽^	149.2 ± 16.3*^,△^	<0.001
Diastolic BP (mmHg)	71.5 ± 8.2^#,▽^	83.9 ± 10.7^*,△,▽^	72.7 ± 8.4^#,▽^	90.2 ± 13.3*^,#,△^	<0.001
Heart rate (bpm)	77.6 ± 13.6	79.3 ± 12.1	78.5 ± 13.0	80.5 ± 13.3	0.129
24-h BP measurement					
Systolic BP (mmHg)	110.0 ± 7.4^#,△,▽^	115.2 ± 6.4*^,△,▽^	122.1 ± 10.2*^,#,▽^	130.0 ± 11.3*^,#,△^	<0.001
Diastolic BP (mmHg)	66.9 ± 5.0^#,△,▽^	69.0 ± 4.8*^,△,▽^	74.1 ± 4.8*^,#,▽^	77.3 ± 6.7*^,#,△^	<0.001
Heart rate (bpm)	70.6 ± 7.3^△,▽^	70.9 ± 7.4	72.3 ± 8.4*	72.3 ± 7.9*	0.022
Daytime BP measurement					
Systolic BP (mmHg)	111.7 ± 8.0^#,△,▽^	117.2 ± 7.2*^,△,▽^	122.4 ± 10.2*^,#,▽^	130.7 ± 11.4*^,#,△^	<0.001
Diastolic BP (mmHg)	68.3 ± 5.4^#,△,▽^	70.6 ± 5.6*^,△,▽^	74.5 ± 5.1*^,#,▽^	78.1 ± 7.1*^,#,△^	<0.001
Heart rate (bpm)	72.9 ± 7.5	73.1 ± 7.8	74.2 ± 8.6	74.3 ± 8.1	0.118
Nighttime BP measurement					
Systolic BP (mmHg)	104.3 ± 7.6^#,△,▽^	108.2 ± 6.6*^,△,▽^	121.0 ± 12.6*^,#,▽^	128.0 ± 14.6*^,#,△^	<0.001
Diastolic BP (mmHg)	62.5 ± 5.0^△,▽^	63.7 ± 4.5^△,▽^	72.8 ± 5.9*^,#^	74.5 ± 7.5*^,#^	<0.001
Heart rate (bpm)	62.9 ± 8.5^△,▽^	63.5 ± 7.6^△^	65.9 ± 9.3*^,#^	65.3 ± 8.4*	<0.001
Blood pressure variability					
SBP standard deviation	12.6 ± 3.2^#, △, ▽^	13.8 ± 3.2*	13.4 ± 3.5*^,▽^	14.3 ± 3.5*^,△^	<0.001
DBP standard deviation	8.9 ± 2.3^▽^	9.5 ± 2.8^▽^	9.3 ± 2.4^▽^	10.1 ± 2.7*^,#,△^	<0.001
SBP coefficient of variation	0.11 ± 0.03^#, △^	0.12 ± 0.03*^,△,▽^	0.11 ± 0.03*^,#^	0.16 ± 0.68 ^#^	<0.001
DBP coefficient of variation	0.13 ± 0.04^△^	0.14 ± 0.10^△^	0.13 ± 0.04*^,#^	0.18 ± 0.68	0.024
Heart rate variability					
SDNN	124.8 ± 37.4^▽^	122.3 ± 48.8	119.7 ± 38.8	113.65 ± 35.1*	0.003
MSD	30.1 ± 37.3^#, ▽^	27.1 ± 39.9*	32.9 ± 45.4^▽^	25.5 ± 33.5*^,△^	0.001
RMSSD	61.2 ± 55.4^▽^	58.8 ± 59.5	64.7 ± 61.8^▽^	54.0 ± 49.9*^,△^	0.028
PNN50 (%)	11.9 ± 16.5^#, ▽^	8.9 ± 14.6*^,△^	12.7 ± 19.8^#,▽^	9.5 ± 16.2*^,△^	<0.001

*BP* blood pressure, *NT* normotension, *WCHT* white-coat hypertension, *MHT* masked hypertension, *SHT* sustained hypertension, *MAP* mean arterial pressure, *SDNN* normal-to-normal RR intervals, *MSD* mean standard deviation of RR intervals, *RMSSD* root mean square of successive differences between adjacent RR intervals, *PNN50* percentage of adjacent NN intervals differing by more than 50 ms

*P < 0.05 vs the NT group; ^#^*P* < 0.05 vs the WCHT group; ^△^*P* < 0.05 vs the MHT group;^▽^*P* < 0.05 vs the SHT group

### Heart rate and blood pressure variability

Heart rate variability in the WCHT and SHT groups was significantly different from that in the NT group, but there was no significant difference between the MHT group and the NT group. The indexes of SDNN, MSD, RMSSD, and PNN50 in the SHT group were lower than those in the NT group, but only MSD and PNN50 were lower in the WCHT group (*P* < 0.05, Table 2). Interestingly, MSD, RMSSD, and PNN50 were also lower in the SHT group than in the MHT group, as was PNN50 in the WCHT group (*P* < 0.05, Table 2).

The BP variability in the WCHT, MHT, and SHT groups was higher than that in the NT group, while the BP variability (SBP standard deviation and DBP standard deviation) in the SHT group was significantly higher than that in the MHT group (*P* < 0.01, Table 2). The DBP standard deviation and SBP coefficient of variation were significantly higher in the SHT group than in the WCHT group (10.1 ± 2.7 vs 9.5 ± 2.8, *P* = 0.028; 0.16 ± 0.68 vs 0.12 ± 0.03 mmHg, *P* = 0.001, Table 2). The SBP coefficient of variation and DBP coefficient of variation also differed in the WCHT group compared with the MHT group (0.12 ± 0.03 vs 0.11 ± 0.03, *P* < 0.001; 0.14 ± 0.10 vs 0.13 ± 0.04 mmHg, *P* = 0.005, Table 2).

### Coronary artery stenosis

The chi-squared test showed that the incidence of CAS in the MHT and SHT groups was significantly higher than that in the NT group, but there was no significant difference between the WCHT group and the NT group (*P* = 0.003, *P* < 0.001, *P* = 0.119, Table 1, Fig. 1b). After adjusting for confounding factors such as gender, age, BMI, diabetes, hyperuricemia, hyperlipidemia, smoking history, and drinking history, the multiple logistic regression analysis showed that age, gender, diabetes mellitus, and hyperlipidemia were independent risk factors for CAS in the WCHT + NT group, while WCHT was not statistically associated with the occurrence of CAS. In the MHT + NT and SHT + NT groups, age, gender, diabetes mellitus, hyperlipidemia, MHT, and SHT were independent risk factors for CAS. All of the logistic regression models showed high goodness of fit (Hosmer–Lemeshows test, *P* > 0.05, Table 3). The one-way analysis of variance showed that Lg (Gensini score) values in the WCHT, MHT, and SHT groups were significantly higher than that in the NT group (1.20 ± 0.51 vs 0.96 ± 0.53, *P* = 0.001; 1.10 ± 0.58 vs 0.96 ± 0.53, *P* = 0.020; 1.30 ± 0.56 vs 0.96 ± 0.53, *P* < 0.001, Table 4, Fig. 1c). Multiple linear regression adjusted for confounding factors such as gender, age, BMI, diabetes, hyperuricemia, hyperlipidemia, smoking history, and drinking history showed persisting significant differences in Lg (Gensini score), and the detailed results are shown in Table 4.

**Table 3:** Logistic regression analysis of CAS incidence between groups

Groups	Parameters	Logistics regression analysis			Hosmer–Lemeshows test
		OR	95% CI	*P* value	*P* value
WCHT versus NT	WCHT	–	–	0.722	0.933
	Gender	0.495	0.330–0.741	0.001	
	Age	2.209	1.669–2.923	<0.001	
	Diabetes	0.171	0.070–0.417	<0.001	
	Hyperlipidemia	1.507	1.008–2.255	0.046	
MHT versus NT	MHT	1.521	1.017–2.276	0.041	0.930
	Gender	0.426	0.291–0.625	<0.001	
	Age	2.183	1.683–2.830	<0.001	
	Diabetes	0.312	0.156-0.623	0.001	
	Hyperlipidemia	1.759	1.199–2.582	0.004	
SHT versus NT	SHT	1.638	1.066–2.516	0.024	0.928
	Gender	0.438	0.297–0.645	<0.001	
	Age	2.088	1.602–2.721	<0.001	
	Diabetes	0.461	0.238–0.894	0.022	
	Hyperlipidemia	1.671	1.129–2.472	0.010	

*NT* normotension, *WCHT* white-coat hypertension, *MHT* masked hypertension, *SHT* sustained hypertension, *CAS* coronary artery stenosis, *OR* odd ratio, *CI* confidence interval

**Table 4:** Comparison of degree of coronary artery stenosis in different hypertension subtypes

Lg (Gensini score)	NT	WCHT	MHT	SHT	*P* -value^*^	*P* value^**^	*P* value^***^
One-way analysis of variance Mean ± SD							
	0.96 ± 0.53	1.20 ± 0.51	1.10 ± 0.58	1.30 ± 0.56	0.001	0.020	<0.001
Multiple linear regression analysis *β* (SE)							
Unadjusted model	–	0.200 (0.068)	0.124 (0.060)	0.301 (0.060)	0.001	0.021	<0.001
Adjusted model	–	0.197 (0.065)	0.100 (0.057)	0.302 (0.059)	<0.001	0.049	<0.001

*NT* normotension, *WCHT* white-coat hypertension, *MHT* masked hypertension, *SHT* sustained hypertension, *Lg (Gensini score)* the logarithm of Gensini score, *SD* standard deviation, *β* standardized regression coefficient, *SE* standard error

*P value, WCHT versus NT; **P value, MHT versus NT; ***P value, SHT versus NT

In view of that result of the IDOCO, Spanish Registry and Jackson Heart Study, it was found that the effects of WCHT on cardiovascular injury were different under various diagnostic criteria. We also performed a statistical analysis of WCHT defined by normal daytime BP and WCHT defined by normal 24-h BP. Under diagnostic criteria A, the incidence of CAS in patients with WCHT defined by normal daytime BP was higher than that in NT patients (71.2 vs 62.5%, *P* = 0.047), and Lg (Gensini score) was also significantly different (1.25 ± 0.53 vs 1.00 ± 0.54, *P* *<* 0.001). Under diagnostic criteria B, Lg (Gensini score) in patients with WCHT defined by normal 24-h BP was higher than that in NT patients (1.25 ± 0.54 vs 0.99 ± 0.53, *P* < 0.001); however, there was no difference in the incidence of CAS between groups (68.3 vs 62.5%, *P* = 0.148).

### Cardiac arrhythmia

The incidences of frequent atrial premature beats, atrial tachycardia, and ventricular arrhythmia in the WCHT and SHT groups were significantly higher than those in the NT group, and the incidence of ventricular arrhythmia in the MHT group was significantly higher than that in the NT group (*P* < 0.05, Table 5). After excluding the patients with CAS, the differences in ventricular arrhythmias between the WCHT, MHT, and SHT groups and NT group disappeared, but the differences in frequent atrial premature beats between the WCHT group and the NT group persisted, as did the differences in frequent atrial premature beats and atrial tachycardia between the SHT group and the NT group (Table 5). After adjusting for gender, age, BMI, diabetes, hyperuricemia, hyperlipidemia, smoking history, and drinking history by logistic regression analysis, the difference in frequent atrial premature beats existed between the WCHT group and the NT group (*P* = 0.028, OR = 2.384, 95% CI: 1.097–5.182), and the incidence of frequent atrial premature beats and atrial tachycardia was also significantly different between the SHT group and the NT group in the subjects with normal coronary arteries (*P* = 0.003, OR = 3.152, 95% CI: 1.488–6.679; *P* = 0.002, OR = 3.267, 95% CI: 1.543–6.916).

**Table 5:** Cardiac arrhythmia incidence of different hypertension subtypes in all subjects and subjects with normal coronary arteries

Parameters	NT	WCHT	MHT	SHT	*P* value*	*P* value**	*P* value***
Total subjects (*N* = 844)							
Frequent atrial premature beats (%)	117 (33.0)	59 (49.6)	76 (39.0)	73 (41.7)	0.001	0.157	0.048
Atrial tachycardia (%)	114 (32.1)	52 (43.7)	73 (37.4)	72 (41.1)	0.022	0.207	0.041
Atrial fibrillation (%)	17 (4.8)	2 (1.7)	15 (7.7)	7 (4.0)	0.135	0.164	0.681
Ventricular arrhythmias (%)	97 (27.3)	46 (38.7)	70 (35.9)	63 (36.0)	0.020	0.036	0.041
Subjects with normal coronary arteries (*N* = 288)							
Frequent atrial premature beats (%)	37 (25.0)	19 (47.5)	20 (35.7)	22 (50)	0.006	0.128	0.002
Atrial tachycardia (%)	36 (24.3)	13 (32.5)	20 (35.7)	22 (50)	0.296	0.104	0.001
Atrial fibrillation (%)	7 (4.7)	2 (5)	3 (5.4)	4 (9.1)	0.943	0.853	0.584
Ventricular arrhythmias (%)	32 (21.6)	14 (35)	18 (32.1)	63 (29.5)	0.081	0.119	0.276

*NT* normotension, *WCHT* white-coat hypertension, *MHT* masked hypertension, *SHT* sustained hypertension

*P value, WCHT versus NT; **P value, MHT versus NT; ***P value, SHT versus NT

## Discussion

There are many limitations in predicting cardiovascular risk solely from clinic BP. Single clinic BP measurement does not reflect the existence of WCHT and MHT [30]. Whether WCHT and MHT involve cardiovascular damage has become a research hotspot. At present, the cardiovascular damage of MHT seems to be established, but the cardiovascular damage of WCHT remains unclear. Much of the research has only used daytime or 24-h ambulatory BP to define WCHT and MHT, whereas our results showed that the proportion of WCHT and MHT changed significantly after adding the nocturnal diagnostic criteria. The proportion of WCHT decreased, while that of MHT increased. Significant changes in hypertension phenotype proportions would also lead to changes in study results. The new diagnostic criteria focus on the ascription of nocturnal hypertension, for example, no longer classifying isolated nocturnal hypertension with cardiovascular damage as NT but as a form of MHT, which we consider reasonable [12, 31]. Exploring the cardiovascular damage of different hypertension phenotypes under the new diagnostic criteria is of great clinical significance.

The cardiovascular injury effect of SHT was relatively positive irrespective of the diagnostic criteria used [32, 33]. SHT has the clinical characteristics of elevated clinic and ambulatory BPs and is clearly associated with other cardiovascular injury risk factors. This study found that SHT is associated with obesity, hyperlipidemia, hyperglycemia, hyperuricemia, and many other cardiovascular risk factors. Changes in heart rate and BP variability are considered to be predictors of cardiovascular events, and SHT has both of these characteristics, mainly manifested as increased cardiac sympathetic nerve function, decreased vagus nerve function and increased BP fluctuations, which are important risk factors for CAS [34–36]. Meanwhile, a higher incidence of CAS in patients with SHT and a worse degree of stenosis was found in this study. MHT also played a corresponding role in coronary artery injury. After adding nighttime diagnostic criteria, the proportion of MHT changed significantly (2.8 to 6.0 to 20.7%). Isolated nocturnal hypertension with cardiovascular damage was redefined as MHT, and the NT subjects were rescreened to increase the likelihood of the NT group being a control group without cardiovascular damage. Cardiovascular damage caused by MHT was reflected in the new diagnostic criteria. We found that MHT was characterized by increased BMI, total cholesterol, triglyceride, and blood glucose and by changes in BP variability but not by clinical features of elevated uric acid and heart rate variability. In addition, BP and heart rate variability were significantly weaker in the MHT group than in the SHT group. Hence, MHT could injure coronary arteries, but not as significantly as SHT.

Cardiovascular damage induced by WCHT has been a clinical research hotspot in the field of hypertension [37, 38]. In particular, the addition of nighttime diagnostic criteria redefined nocturnal hypertension with elevated clinic BP as SHT, while the proportion of WCHT was correspondingly reduced. Faria Joao et al. did not find an increased incidence of cardiovascular events in patients with nocturnal NT WCHT during a 7.6-year follow-up study [39]. However, abnormal glucose and lipid metabolism and cardiac structural changes in nocturnal normal WCHT have also been reported in many studies [12, 40]. Whether WCHT also causes cardiovascular damage remains controversial. This study found no association between WCHT and the incidence of CAS. However, the incidence of CAS was higher in patients with WCHT than in the control group when only daytime BP criteria were used. Consistent with the research results of IDOCO et al., the effect of WCHT defined by normal daytime BP on coronary artery injury should be more significant than that of WCHT defined by normality of all periods. However, this did not mean that WCHT defined by normality of all periods did not cause coronary artery injury, since numerous cardiovascular injury factors, such as triglyceride, blood glucose, BP variability, and heart rate variability, were associated with WCHT defined by normality of all periods in this study. We also found that WCHT defined by normality of all periods was significantly correlated with the severity of CAS, and after adjustment for age, gender, and other confounding factors by multiple linear regression, the significant difference persisted. WCHT probably remains a phenotype of hypertension with coronary artery injury, diagnosed with the addition of nighttime BP criteria.

Previous studies have suggested that the arrhythmia-induced effects of hypertension are mainly related to cardiac hypertrophy, and in the Framingham Heart Study, echocardiographic evidence of LVH was proven to be associated with ventricular arrhythmias. In this study, we further explored the relationship between hypertension and cardiac arrhythmias based on two aspects. First, we classified hypertension into three phenotypes: WCHT, MHT, and SHT. Second, the interference of CAS on the study results was fully considered. The incidence of atrial arrhythmias, such as frequent atrial premature beats, was higher in the WCHT and SHT groups than in the NT group, but no such changes were found in the MHT group. Heart rate variability is an important indicator of cardiac autonomic nervous function and is closely related to the occurrence of cardiac arrhythmias. Heart rate variability changes were found in the WCHT and SHT groups compared with the NT group, and these changes were characterized by increased sympathetic excitability and decreased vagal excitability, while no corresponding changes were found in the MHT group. The differences in cardiac autonomic nervous function in hypertension phenotypes may lead to the different effects of atrial arrhythmias caused by different hypertension phenotypes. In addition, the WCHT, MHT, and SHT groups showed increased ventricular arrhythmias, but this difference disappeared in the participants with normal coronary arteries, indicating that CAS interfered with the results of the study. Ventricular arrhythmias in hypertension phenotypes may be related to the increased severity of CAS in patients with WCHT, MHT, and SHT.

This study aimed to investigate whether hypertension phenotypes such as WCHT, diagnosed by 24-h BP, daytime BP, and nighttime BP, were related to CAS and cardiac arrhythmia. The cardiovascular injury effects of MHT and SHT were further verified. Although no correlation was found between WCHT and the occurrence of CAS, WCHT was related to the aggravation of CAS and the occurrence of cardiac arrhythmia. Hence, WCHT cannot be treated as a benign hypertension phenotype. The advantage of this study was that the strict control of exclusion criteria to exclude users of antihypertensive drugs, lipid-lowering drugs and/or antiplatelet drugs increased the credibility of the study sample. However, this study had some limitations. First, this was a cross-sectional observational study. Hence, the risk of cardiovascular events and clinical prognosis of different hypertension phenotypes under the new diagnostic criteria need to be verified by long-term follow-up studies. Second, given the sufficient clinical evidence that hypertension phenotypes can cause cardiac structural changes, echocardiographic data such as left ventricular mass index were not collected, and the effect of hypertension phenotypes such as WCHT on cardiac structure was not explored in this study.
